# Beyond the Tipping Point: Advances in the Diagnosis and Management of Acute-on-Chronic Liver Failure and End-Stage Liver Disease

**DOI:** 10.3390/diagnostics16101548

**Published:** 2026-05-20

**Authors:** Jonathan Soldera

**Affiliations:** 1 PGDip and MSc Acute Medicine and Gastroenterology, University of South Wales in Association with Learna Ltd., Cardiff CF37 1DL, UK; jonathansoldera@gmail.com; 2 Gastroenterology, Logan Hospital, Brisbane, QLD 4131, Australia

**Keywords:** acute-on-chronic liver failure, liver cirrhosis, hepatorenal syndrome, liver transplantation, end stage liver disease

## Abstract

Acute-on-chronic liver failure (ACLF) is the point at which cirrhosis stops behaving as a chronic liver disease and becomes a rapidly destabilising systemic illness. It is the real tipping point in advanced liver disease: the moment when limited hepatic reserve is no longer the only issue, and the clinical picture is instead defined by systemic inflammation, extrahepatic organ dysfunction, and a high risk of short-term death. This has changed how we understand the natural history of cirrhosis. Rather than a simple linear progression toward liver failure, advanced chronic liver disease is now better seen as a dynamic continuum that may lead to first decompensation, recurrent decompensation, ACLF, end-stage disease, or, in selected cases, recompensation if the underlying driver is effectively controlled. This shift matters because patients with ACLF are not simply “sicker cirrhotics”. They are in a distinct pathophysiological state, marked by inflammation, circulatory dysfunction, immune dysregulation, and organ cross-talk that extends beyond the liver. In this setting, the boundaries between liver failure, sepsis, renal dysfunction, and critical illness become blurred, which is why ACLF remains such a difficult syndrome to manage. At the same time, recent guidance has improved the approach to decompensated cirrhosis, HRS-AKI, infection, transplantation, and palliative care, while newer consensus efforts have tried to reduce differences between ACLF definitions. In practice, management still depends on simple but disciplined principles: early recognition, rapid identification of precipitants, parallel organ support, prompt treatment of infection and HRS-AKI, repeated reassessment, and urgent transplant evaluation when appropriate. This review examines ACLF and end-stage liver disease as interconnected stages of advanced cirrhosis and discusses how care can be both aggressive when recovery is possible and humane when recovery is not.

## 1. Introduction: The Modern Tipping Point in Cirrhosis

End-stage liver disease (ESLD) is no longer best understood as the final step of a simple linear descent from compensated cirrhosis to terminal liver failure. The contemporary view is more dynamic and clinically more useful. Portal hypertension, systemic inflammation, precipitating insults, and residual organ reserve interact continuously to determine whether a patient remains stable, develops a first decompensation, enters recurrent instability, recompensates after control of the underlying driver, or progresses to acute-on-chronic liver failure (ACLF) [1,2,3,4]. Within this framework, ACLF should not be reduced to the idea of “very sick cirrhosis.” It is a distinct syndrome of acute decompensation complicated by organ failure and marked by a disproportionate risk of short-term death, a point consistently supported by the EASL-CLIF literature and by the prognostic performance of CLIF-based models [5]. At a population level, this is not a marginal phenotype: among patients hospitalized with decompensated cirrhosis, ACLF accounts for a substantial proportion of admissions and carries very high short-term mortality, although both prevalence and outcome vary by region, underlying liver disease, and precipitating events [6].

**Figure 1:**
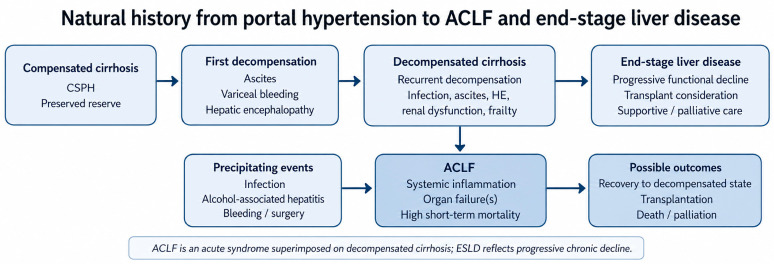
Natural history from Portal Hypertension to ACLF and ESLD.

The distinction matters because it changes priorities at the bedside. In compensated cirrhosis, the dominant objective is to prevent first decompensation through etiologic treatment, portal pressure reduction, and better risk stratification. Once decompensation has occurred, however, subsequent events no longer carry the same biological meaning they did earlier in the disease course. Ascites, infection, bleeding, and encephalopathy are not merely complications to be listed; they often reflect progressive exhaustion of reserve. In a subset of patients, a further inflammatory, infectious, or haemodynamic insult is enough to shift the clinical picture from decompensated cirrhosis to multiorgan failure. That is the point at which ACLF becomes most relevant. In real-world cohorts, ACLF has been independently associated with worse survival in patients admitted with spontaneous bacterial peritonitis [7] and in those presenting with acute esophageal variceal hemorrhage [8]. Its prognostic weight is especially evident when bacterial infection is the precipitating event, as infection-related ACLF carries a particularly severe course, high rates of organ failure progression, and worse short-term survival than many non-infectious triggers [9,10]. Therefore, although ACLF and ESLD are not interchangeable—ACLF reflecting acute organ-failure-driven instability and ESLD the broader state of advanced liver disease with limited reserve and high symptom burden—their overlap becomes especially evident when infection pushes decompensated cirrhosis from chronic fragility into systemic critical illness [11,12,13] (Figure 1).

Not all infections behave the same way: infection-related ACLF varies by geography, microbiology, and infectious source; spontaneous bacterial peritonitis, pneumonia, multidrug-resistant organisms, and delayed antibiotic therapy are all associated with worse trajectories in different cohorts [11,12,13]. This is precisely why the management of cirrhosis can no longer be framed only in terms of hepatic reserve or portal hypertension. Early recognition of sepsis, prompt source control, timely antibiotics, vasopressor support, and careful haemodynamic assessment are now part of hepatology as much as endoscopy, paracentesis, or transplant referral [8]. Any contemporary review of ACLF and ESLD therefore has to connect definition with management, prognosis with trajectory, and organ support with decisions about reversibility, integrating Hepatology, Intensive Care and Palliative Care [14,15].

The aim of this review is to examine, in an integrated and clinically grounded manner, how current concepts of portal hypertension, acute decompensation, systemic inflammation, infection, extrahepatic organ failure, prognostic stratification, liver transplantation, and palliative care intersect in the modern understanding of ACLF and ESLD, and how these advances can be translated into earlier recognition, better bedside decision-making, and more realistic and humane care for patients who have moved beyond the tipping point of cirrhosis.

## 2. From Compensated Cirrhosis to Decompensation and Recompensation

The modern understanding of cirrhosis rests on a shift from a static, morphology-based description of liver disease to a dynamic clinical model centered on portal hypertension, systemic inflammation, and physiological reserve. The 2018 EASL guidance on decompensated cirrhosis and the Baveno VII consensus remain the main pillars of this framework [1,2]. In practical terms, clinically significant portal hypertension remains the principal driver of the classic complications of cirrhosis, particularly ascites, variceal hemorrhage, and hepatic encephalopathy, and the first decompensating event remains the major transition point in prognosis in the course of the disease [1,2]. Once that threshold is crossed, survival declines sharply and the patient enters a less stable phase in which subsequent events no longer behave as isolated complications, but as markers of diminishing reserve and increasing biological fragility.

There is also an important historical perspective. Over the last four decades, survival in cirrhosis has improved in a way that appears to reflect genuine advances in hepatology care rather than merely differences in the patients being hospitalized. Endoscopic hemostasis, vasoactive therapy, antibiotic prophylaxis in bleeding, albumin-based strategies, safer paracentesis, more structured management of portal hypertension, better intensive care, and transplantation have all changed outcomes meaningfully. Population-level data from the United States showed that inpatient mortality among hospitalized patients with cirrhosis fell substantially between 2002 and 2010 despite increasing age and comorbidity, supporting the view that advances in cirrhosis care were translating into real survival benefit [16]. Earlier work focused on preserving patients with advanced decompensation in transplantable condition had already made a similar point: in cirrhosis, supportive care is not merely adjunctive but can itself become a survival strategy [17].

That progress, however, has been uneven. Hepatology has become much better at controlling many of the classic complications of portal hypertension than at reversing the syndromes driven by infection, systemic inflammation, and circulatory dysfunction. Ascites can often be temporized, variceal bleeding is managed far more effectively than it once was, and encephalopathy is usually treatable. By contrast, infection and hepatorenal dysfunction continue to define some of the most dangerous turning points in advanced cirrhosis. Although mortality associated with several complications declined over time, sepsis remained a particularly powerful determinant of death and became relatively more prominent as other aspects of care improved [16]. More recent work has reinforced that bacterial infection is not a peripheral complication in advanced liver disease, but one of the main mechanisms through which decompensated cirrhosis progresses toward organ failure [16,17,18,19,20]. This is why prevention of decompensation has become a major therapeutic goal in its own right. Portal pressure reduction, disease-modifying pharmacologic strategies, and earlier treatment of the underlying liver disease all reflect a broader shift from reacting to complications toward preventing the first clinical collapse [21]. Importantly, Baveno VII introduced the concept of recompensation, making clear that decompensated cirrhosis is not always a strictly one-way trajectory, although such recovery requires durable etiologic control and remains the exception rather than the rule [2,22,23,24].

Still, recompensation should not be overstated. For most patients, decompensation marks the beginning of recurrent instability rather than recovery. Recurrent ascites, hyponatremia, sarcopenia, infection, renal dysfunction, and encephalopathy usually reflect progressive exhaustion of reserve rather than separate and unrelated events. It is within this population that ACLF most often emerges, either at admission or early during hospitalization [3,4]. In clinical practice, patients who progress from decompensation to ACLF are often those in whom the next insult exceeds the stabilizing effect of modern cirrhosis care, especially when severe infection, worsening circulatory dysfunction, or HRS-AKI are present. This pattern is increasingly reflected in contemporary data. Infection-related ACLF is common, regionally variable, and associated with particularly poor outcomes; resistant organisms, septic shock, and delayed effective treatment all worsen the trajectory once decompensation has tipped into organ failure [19,20,25,26]. This ACLF scenariowith higher CLIF-SOFA scores has been independently associated with worse survival, reinforcing that once organ failure emerges, prognosis changes sharply [27,28,29].At the same time, the course after listing for transplantation and the limitations of MELD-based allocation conveys that once ACLF supervenes, short-term risk is not fully captured by chronic disease severity alone [30,31,32,33,34,35]. That tension defines the current era of cirrhosis care: we have become considerably better at treating many manifestations of decompensation, but infection, renal dysfunction, and the inflammatory-hemodynamic cascade that links them to ACLF remain the clearest expression of where present treatment still reaches its limit.

## 3. Defining ACLF: Why Geography Still Matters

One of the persistent difficulties in the ACLF literature is that, despite the maturity of the field, ACLF is still not a universally defined syndrome. This is not a trivial taxonomic problem. The way ACLF is defined determines which patients are identified, how prognosis is estimated, who enters clinical studies, and how results are interpreted across regions and healthcare systems. The CANONIC study and the subsequent EASL-CLIF framework marked a decisive step because they shifted the discussion away from vague “acute worsening” and toward a syndrome clinicians could recognize at the bedside: acute decompensation of cirrhosis accompanied by organ failure and associated with high short-term mortality [3]. That approach has become particularly influential because it captures what is often most decisive in practice, namely the emergence of extrahepatic organ dysfunction and the abrupt change in short-term prognosis that follows. It is also the most extensively studied framework in the contemporary literature and the one that has generated the largest body of prognostic and translational work [4,36,37,38].

In practical terms, EASL-CLIF defines ACLF in hospitalized patients with cirrhosis who develop acute decompensation and organ failure, graded according to the type and number of organ failures using the CLIF-C Organ Failure system [3,4]. This makes it especially useful for patients in whom renal, circulatory, cerebral, respiratory, or coagulation dysfunction has become the dominant clinical problem. That strength is reflected in the performance of CLIF-based tools across multiple cohorts. CLIF-SOFA, CLIF-C ACLF, and CLIF-C AD have repeatedly shown strong prognostic performance in ACLF and acute decompensation, generally outperforming older liver-specific models once multiorgan dysfunction becomes the central issue [4]. This pattern is supported by cohort data in decompensated cirrhosis and ACLF, where CLIF-SOFA and CLIF-C scores outperformed other traditional liver scores in various scenarios [27,28,29,39,40,41,42]. Direct comparisons have also strengthened the case for EASL-CLIF. In large cohorts, EASL-CLIF criteria identify substantially more patients with clinically meaningful ACLF than NACSELD and provide better granularity for short-term prognostication, particularly when organ failure burden is escalating [36,37,43]. Even in non-transplant settings, EASL-CLIF tends to capture the broader clinically relevant population, whereas narrower definitions may identify fewer but often more advanced cases [43].

APASL developed from a different conceptual starting point. Historically, it defined ACLF as an acute hepatic insult in a patient with chronic liver disease or cirrhosis, characterized by jaundice and coagulopathy complicated within 4 weeks by ascites and/or encephalopathy. The emphasis is therefore more hepatic than extrahepatic at entry, and the acute insult itself occupies a more central place in the syndrome definition. This reflects, at least in part, the epidemiology and case mix seen in much of Asia, where hepatitis B reactivation, alcohol-related liver injury, and other primary hepatic insults have played a larger role than in many Western cirrhotic cohorts [44,45,46,47,48]. The APASL framework has practical value because it is easy to apply, captures a clinically recognizable syndrome, and remains highly relevant in regions where acute hepatic injury is the dominant precipitating event. It has also generated its own prognostic ecosystem, particularly through the AARC model, which performs well within APASL-defined cohorts, especially when assessed dynamically over the first week [47]. At the same time, APASL does not identify exactly the same patients as EASL-CLIF. Comparative studies from Asia have shown that APASL may classify a broader group at an earlier hepatic-injury stage, while EASL-CLIF more clearly separates those with extrahepatic organ failure and worse short-term prognosis [49,50]. The more recent harmonization efforts and the Kyoto Consensus are important precisely because they acknowledge these differences rather than pretending they do not exist [44,48].

NACSELD occupies yet another position. Developed in North American hospitalized cohorts, it defines ACLF more narrowly through the presence of multiple extrahepatic organ failures in cirrhotic patients, often in the setting of infection, and was designed with a strong focus on short-term inpatient survival [35,51]. Its appeal lies in its simplicity. It functions well as a pragmatic bedside tool and has been externally validated in large national cohorts for short-term survival prediction [35]. In some settings, particularly for in-hospital mortality, the presence of NACSELD-ACLF performs at least as well as more complex grading systems [35,45]. But simplicity comes with trade-offs. NACSELD identifies a smaller population than EASL-CLIF, is less sensitive for earlier or intermediate stages of ACLF and tends to capture a later and more selective phenotype of organ failure [36,37,43]. In other words, it is useful, but it is not equivalent. A patient may already be in clinically meaningful ACLF by EASL-CLIF criteria while still not meeting NACSELD criteria. That matters not only for prognosis, but for escalation decisions, trial eligibility, and transplant timing.

For that reason, the differences among EASL-CLIF, APASL, and NACSELD should not be treated as mere academic fragmentation. They define different entry points into the same dangerous territory. EASL-CLIF is centered on acute decompensation plus organ failure in cirrhosis; APASL is centered on acute hepatic insult with jaundice and coagulopathy in chronic liver disease; NACSELD is centered on extrahepatic organ failure in hospitalized cirrhotic patients, often with infection [43]. These different starting points produce different epidemiology, different patient selection, and different prognostic behavior. But for defining ACLF itself in hospitalized cirrhotic patients with multiorgan dysfunction, EASL-CLIF remains the most comprehensive and most clinically useful framework.

Clinicians should therefore avoid the following two errors: pretending that one definition has made all the others irrelevant, or pretending that they are interchangeable. *They are not*. APASL and NACSELD remain important because they reflect different biological emphases, different clinical populations, and different healthcare realities [43,44,48,51]. EASL-CLIF has become the dominant reference standard in the contemporary literature because it best captures the syndrome most hepatologists and intensivists confront when acute decompensation evolves into multiorgan failure, and because its derived scores have shown the most consistent utility for severity stratification, prognostication, and transplant-oriented decision-making [4,35,36,37,43]. Geography still matters because disease biology, precipitating events, and systems of care still matter. The task is not to force artificial uniformity, but to understand clearly what each framework identifies, what it excludes, and how that shapes the interpretation of prognosis, trials, and bedside decisions (Table 1).

## 4. Pathophysiology: Systemic Inflammation as the Final Common Pathway

Portal hypertension, hepatocellular dysfunction, and reduced physiological reserve create the substrate on which ACLF develops, but they do not by themselves explain the abrupt transition from chronic instability to multiorgan failure. The pathophysiologic model that best accounts for that transition is systemic inflammation. In ACLF, a precipitating event does not simply worsen liver tests; it activates an already primed inflammatory network that extends beyond the liver and begins to disrupt the function of multiple organs at once [3,4,52]. This is why ACLF is better understood as a syndrome of acute systemic decompensation occurring in advanced chronic liver disease than as a severe hepatic insult alone. The inflammatory state is rarely driven by a single mechanism. It reflects the convergence of pathogen-associated molecular patterns derived from bacterial translocation, damage-associated molecular patterns released from injured tissue, alcohol-related hepatitis, overt bacterial infection, ischemia-reperfusion phenomena, oxidative stress, and sterile inflammatory signaling, all acting on an immune system that is already dysregulated by cirrhosis and ESLD [4,20,39,52,53].

The gut–liver axis sits at the center of this process. Increased intestinal permeability, bacterial overgrowth, dysbiosis, and sustained bacterial translocation expose the portal and systemic circulation to persistent inflammatory stimulation. This does not result merely in “more liver injury.” It produces a syndrome of systemic endothelial activation, microcirculatory failure, nitric oxide-mediated vasodilatation, mitochondrial dysfunction, impaired oxygen utilization, and progressive loss of effective perfusion [39,53]. At that stage, the severity of illness is no longer explained by hepatic insufficiency alone. That is why extrahepatic organ failures, particularly renal, cerebral, circulatory, and respiratory failures, strongly determine short-term outcomes in ACLF [3,4]. The same framework also links ACLF to ESLD more broadly. In ESLD, chronic portal hypertension, recurrent decompensation, immune dysfunction, oxidative stress, and circulatory derangement create the biological terrain on which a further inflammatory hit can trigger abrupt collapse [54,55]. In that sense, ACLF is not separate from ESLD pathobiology; it is its most unstable and inflammatory expression.

This inflammatory model is also increasingly supported by biomarker data. Conventional liver scores capture hepatic reserve, but they do not fully capture the intensity of systemic inflammation or the host response to injury. Markers of macrophage activation, neutrophil injury, oxidative albumin damage, and inflammatory signaling improve prognostic discrimination when added to standard clinical models, reinforcing that inflammation is not merely associated with ACLF, but mechanistically central to its development and progression [52,56,57]. Oxidative modification of albumin is particularly relevant in advanced chronic liver failure and/or sepsis, because it links oxidative stress, impaired albumin function, circulatory dysfunction, and survival [57]. Likewise, cytokine pathways such as TGF-β1/IL-31 in HBV-related ACLF suggest that the inflammatory cascade is not biologically generic, but composed of specific signaling networks that may carry prognostic and, eventually, therapeutic relevance [55]. The broader message is that ACLF behaves less like a pure failure of hepatic synthetic function and more like a systemic syndrome in which inflammation amplifies organ vulnerability and accelerates collapse.

Within this framework, the cardiorenal axis deserves particular emphasis. In advanced cirrhosis, circulatory dysfunction is not limited to splanchnic vasodilatation and effective hypovolemia. A more complex interaction develops between systemic inflammation, neurohumoral activation, portal hypertension, and impaired cardiac reserve. Cardiac output may initially be preserved or even increased, but as disease advances, contractile reserve falls and the ability to maintain organ perfusion under stress becomes limited. This is particularly relevant during infection or acute decompensation, when an adequate cardiac response is needed to preserve renal blood flow. When that compensatory response fails, renal dysfunction and hepatorenal syndrome can follow, not as isolated renal events, but as part of a broader cardiorenal interaction in which haemodynamic failure, inflammatory activation, and myocardial dysfunction converge [58,59]. Portal and systemic haemodynamic derangements are not simply epiphenomena in this setting; they are strongly linked to both mortality and reversibility, and improvement in those parameters parallels recovery in patients who survive the acute phase. Even in ESLD outside the classic ACLF setting, systemic vasodilatation and altered vascular reactivity remain central physiologic features, reinforcing how closely portal, systemic, cardiac, and renal dysfunction are intertwined in advanced liver disease [58].

**Figure 2:**
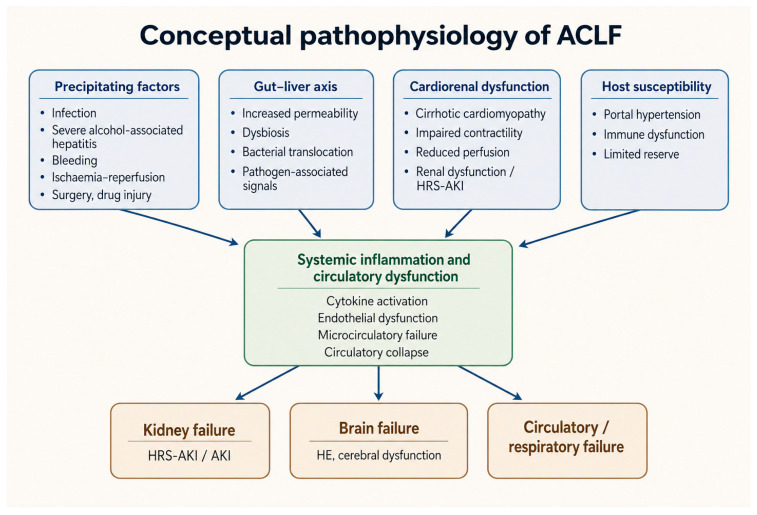
Conceptual pathophysiology of ACLF.

This also explains why infection is such a potent precipitant of ACLF and renal failure. Patients with cirrhosis already exist in a state of chronic low-grade inflammation, immune paresis, and endotoxemia. When bacterial infection supervenes, the host response resembles sepsis and septic shock, with further cytokine activation, vasodilatation, tissue hypoperfusion, and worsening circulatory stress [7,8,39]. In this context, myocardial dysfunction, impaired vascular responsiveness, and renal hypoperfusion amplify one another, turning a treatable infection into a multiorgan syndrome. This is why infection-related ACLF is so biologically aggressive and why timing matters so much. The syndrome is not defined only by the microorganism, but by the host response it starts [7,8,39]. The same logic applies to other acute insults, including alcohol-related hepatitis, drug-induced liver injury, acute viral hepatitis, and severe bleeding. What makes them dangerous is not simply that they injure the liver, but that in a patient with ESLD they can ignite an inflammatory and haemodynamic cascade that the system can no longer contain [44,55,60] (Figure 2).

The inflammatory model also helps reconcile an observation familiar to any clinician caring for these patients: two patients with similar MELD or Child-Pugh values may behave very differently. One patient with refractory ascites may remain chronically unwell but relatively stable, while another with comparable laboratory values deteriorates rapidly because systemic inflammation, circulatory dysfunction, and extrahepatic organ failure are actively evolving. For this reason, ACLF cannot be understood through measures of hepatic reserve alone. It requires assessment of organ failure, haemodynamic context, inflammatory burden, and, above all, clinical trajectory over time [3,4,52,56]. Frailty may also belong in this broader pathophysiologic frame. Although it is not a direct inflammatory biomarker, it reflects the host’s functional reserve and influences the capacity to recover from acute systemic stress; dynamic changes in frailty during hospitalization may therefore provide additional information about the reversibility of ACLF biology [61]. Put simply, ACLF is the point at which the pathophysiology of advanced cirrhosis becomes unmistakably systemic. The liver remains central, but it is no longer acting alone.

## 5. Precipitating Events and the Importance of Active Case Finding

ACLF rarely develops without a precipitating event. In most patients, it emerges when an acute insult acts on the background of advanced cirrhosis, portal hypertension, immune dysfunction, and reduced physiological reserve. For that reason, identifying the trigger is not a secondary diagnostic exercise but one of the few parts of ACLF care that can still change trajectory in real time. The major precipitants remain bacterial infection, severe alcohol-associated hepatitis, gastrointestinal bleeding, drug-induced liver injury, and major procedures or surgery, although a substantial minority of patients still present without an immediately obvious cause [4,62]. That absence should not reassure the clinician. It should instead prompt a deliberate search for occult infection, medication-related toxicity, ischemic injury, endocrine or hemodynamic instability, and non-hepatic events such as cardiopulmonary deterioration.

This framework has become more structured over time. The PREDICT study showed that a relatively small group of precipitants accounts for most episodes of acute decompensation and ACLF, particularly proven bacterial infections and severe alcohol-associated hepatitis, either alone or in combination [63]. What matters, however, is not only the type of trigger but the biological load it imposes. Multiple simultaneous insults are associated with greater systemic inflammation and worse outcomes, which is consistent with the broader concept that ACLF is less a single-event syndrome than a collapse produced by cumulative inflammatory and hemodynamic stress in a vulnerable host [63,64,65]. This also helps explain why ACLF often behaves clinically like “sepsis plus liver failure”: the precipitant activates a septic-like inflammatory cascade in a host whose cirrhotic liver can no longer buffer endotoxemia, maintain immune balance, or preserve organ perfusion.

Infection sits at the center of this model. It is one of the most frequent precipitants of ACLF worldwide, one of the strongest determinants of short-term mortality, and one of the clearest examples of how advanced cirrhosis becomes a systemic critical illness rather than a liver-limited disease [11,12,13,66,67,68]. Infection-related ACLF is not biologically uniform. Its presentation varies by geography, microbiology, infectious source, severity of circulatory disturbance, and timing of antimicrobial treatment. Bacterial infection-related ACLF accounts for a large proportion of cases globally, with spontaneous bacterial peritonitis, pneumonia, urinary infection, and multidrug-resistant organisms repeatedly associated with worse outcomes [11,12,66,67,68]. Prognosis also differs by source: spontaneous bacterial peritonitis and bacteremia do not carry the same mortality signal as skin and soft tissue infection, and fungal infection, although less frequent, is associated with particularly poor outcomes once ACLF is established [12,68,69]. In ICU cohorts with alcohol-related cirrhosis, sepsis has again emerged as the predominant precipitating event and the strongest determinant of short-term prognosis, reinforcing that once infection progresses to circulatory collapse, the syndrome becomes qualitatively different from uncomplicated decompensation [18,70].

The reason infection is so potent in cirrhosis is not simply microbial exposure, but cirrhosis-associated immune dysfunction. Patients with advanced cirrhosis and ACLF occupy a paradoxical state of simultaneous hyperinflammation and ineffective host defense. They overproduce inflammatory mediators and yet clear pathogens poorly. This is the logic of immunoparesis. Monocyte deactivation, reduced HLA-DR expression, impaired ex vivo cytokine response, complement dysfunction, altered opsonization, and gut barrier failure all contribute to a state in which the patient is both inflamed and immunocompromised at the same time [71,72,73,74]. That paradox explains why ACLF so often resembles sepsis biologically even when cultures are initially negative. The trigger may be a proven infection, but it may also be endotoxemia, bacterial translocation, or DAMP/PAMP-driven inflammation in a patient whose immune system is already dysregulated. Once this process begins, endothelial dysfunction, vasodilatation, tissue hypoperfusion, renal dysfunction, and multiorgan failure can evolve quickly [64,65,66,75]. Experimental work has further suggested that sepsis-associated endothelial dysfunction is not merely associated with ACLF, but may directly drive the loss of hepatic differentiation and synthetic function through specific endothelial–hepatocyte signaling pathways, underscoring that infection can worsen ACLF through mechanisms beyond conventional tissue injury [66].

For that reason, active case finding is central to management. Diagnostic paracentesis in patients with ascites, blood and urine cultures, chest imaging, early review of invasive lines, medication reconciliation, and a low threshold for cross-sectional imaging are not optional add-ons; they are core elements of care. The management of infection in ACLF is especially time-sensitive because delayed or inadequate empirical antibiotic therapy is consistently associated with worse outcomes [12,25,67,68]. This is one of the few moments in ACLF where acting earlier can change the biology rather than merely document its consequences. Early and appropriate antibiotics reduce progression and mortality, whereas antibiotic delay, multidrug resistance, and failure of source control move the patient rapidly toward shock and organ failure [12,25,63,67]. At the same time, these data argue against simplistic uniformity. In cirrhotic patients with acute variceal bleeding, for example, antibiotic prophylaxis remains standard in the broader population, yet recent data suggest that the risk-benefit balance may differ in carefully selected Child–Pugh A patients [76]. The point is not to weaken vigilance, but to acknowledge that precipitant-specific care is becoming more nuanced.

Alcohol-associated hepatitis occupies a similarly important but more complex position. It is both a precipitating event and, in some patients, an autonomous inflammatory syndrome capable of sustaining organ dysfunction even without another obvious trigger. In patients with recent heavy alcohol exposure, jaundice, worsening coagulopathy, renal dysfunction, and circulatory instability, severe alcohol-associated hepatitis and ACLF often coexist, and the distinction between “trigger” and “phenotype” becomes blurred [63,64,65]. This matters because alcohol-related ACLF is not only common, but also biologically aggressive, often complicated by infection, malnutrition, sarcopenia, and hemodynamic fragility [77,78,79]. In practice, these patients require active parallel evaluation for superimposed bacterial infection rather than assuming that all inflammatory worsening is attributable to alcohol alone.

Other precipitants remain clinically relevant even if they are less dominant in large consortia. Autoimmune liver disease may destabilize through active autoimmune hepatitis or overlap syndromes [80,81,82]. Herb-induced liver injury can precipitate acute decompensation and occasionally ACLF in an already vulnerable liver [83,84,85]. Hepatocellular carcinoma can drive decline through tumour burden, bleeding, infection, malnutrition, or metastatic complications [86,87,88,89,90]. Chronic viral hepatitis also remains relevant because HBV flares can still precipitate acute deterioration, even as successful HCV treatment has changed the long-term trajectory of many patients who would previously have continued to decompensate [91,92]. These triggers do not contribute equally in every region or cohort, but together they reinforce an important principle: ACLF is clinically heterogeneous, and its precipitants reflect both local epidemiology and the biological vulnerabilities of the underlying liver disease.

The importance of active case finding extends beyond the index admission. Kidney dysfunction, for example, should not be viewed only as a downstream complication of established ACLF. Recent data suggesting that baseline renal impairment independently predicts subsequent sepsis support the idea that renal dysfunction can also function as an early marker of systemic vulnerability and future inflammatory decompensation [93]. Likewise, early readmission after discharge is increasingly recognizable as a measurable high-risk state rather than a vague clinical impression, with scores such as REACT helping identify patients with decompensated cirrhosis who are likely to deteriorate again soon after hospitalization [94]. This matters because the first precipitant is often not the last. After an initial decompensation, what happens next strongly shapes prognosis. Further decompensation markedly increases the cumulative incidence of liver-related death or transplantation, whereas etiologic therapy and non-selective beta-blockers appear protective [95]. Seen this way, precipitants are not isolated events but part of a broader sequence of destabilization.

This sequential view also helps explain why timing and order matter. Organ failure that develops during hospitalization may carry a worse prognosis than organ failure already present at admission, and liver failure and renal failure do not appear to have identical temporal or prognostic meanings [96]. In acute variceal bleeding, ACLF clearly identifies a subgroup with substantially worse outcomes, but the relative contributions of bleeding itself, infection, hepatic reserve, and organ failure differ across cohorts [8,97,98]. In spontaneous bacterial peritonitis, nosocomial and community-acquired disease also appear to carry distinct prognostic patterns, arguing against treating all SBP as biologically equivalent [69]. These observations support a more phenotype-specific and time-sensitive model of ACLF prevention and management, in which trigger, host response, sequence of organ failure, and reversibility all matter (Table 2).

Ultimately, identifying the precipitant is not just about naming the cause of deterioration. It is about interrupting the inflammatory cascade before it becomes self-propagating. As mechanistic work from the CANONIC cohort and subsequent immune studies have shown, the intensity of systemic inflammation correlates with the presence, severity, and trajectory of ACLF, but the host response also includes a compensatory anti-inflammatory phase marked by immune exhaustion and impaired pathogen clearance [64,72,73,74,99]. This is why patients with ACLF are so vulnerable to secondary infection and why the syndrome can progress even after the initial trigger appears to have been treated. The bedside implication is simple but unforgiving: if the precipitant is not actively sought, it will be missed; if it is missed, the window to change the course of ACLF may close quickly.

## 6. Advances in Diagnosis and Risk Stratification

Diagnosis in ACLF begins with a simple but decisive premise: acute deterioration in cirrhosis is not a uniform event. A patient admitted with tense ascites, preserved mentation, and stable kidney function does not carry the same biological risk as another with worsening encephalopathy, rising creatinine, hypoxemia, and circulatory failure, even if both are initially described as having decompensated cirrhosis. Modern assessment therefore moved away from liver dysfunction alone and toward structured organ failure profiling. This is the main practical contribution of the EASL-CLIF framework. By combining the presence and severity of organ failures through the CLIF-SOFA score and then refining prognostic assessment with age and white cell count in the CLIF-C models, this framework captures more faithfully the syndrome that determines short-term outcome in ACLF: multiorgan dysfunction occurring in the setting of systemic inflammation [4,5,44].

This shift also corrected an older misconception that ACLF is mainly an ICU curiosity. It is not. Global data show that ACLF affects roughly one-third of patients hospitalized with decompensated cirrhosis, although prevalence varies by geography, underlying disease, and the definition applied [62]. Cohort data have reached similar conclusions, showing that ACLF complicates a substantial proportion of non-elective cirrhosis admissions and identifies patients with markedly worse short-term transplant-free survival [7,8,27,28,29,39,40,41,42,63]. The practical implication is straightforward: ACLF should be looked for early in any acute hospital admission with cirrhosis, not recognized only after organ failure has become advanced and clinically obvious.

From a bedside perspective, the most useful scoring systems are now reasonably clear. For diagnosis and staging of ACLF in hospitalized patients with cirrhosis, the EASL-CLIF approach remains the most clinically informative framework. For prognostication, CLIF-SOFA helps define the burden of organ failure, CLIF-C ACLF is the most useful score once ACLF is established, and CLIF-C AD remains helpful in patients with acute decompensation who have not yet crossed into ACLF [44]. MELD, MELD-Na, and Child-Pugh still retain value for chronic liver disease severity, transplant listing context, and broad cirrhosis assessment, but when extrahepatic organ dysfunction becomes the dominant driver of short-term mortality, CLIF-based scores should take precedence in everyday practice [7,8,27,28,29,39,40,41,42]. Put simply, if the clinical problem is ACLF, the most useful prognostic tools are the ones built around ACLF biology.

This is why CLIF-based models deserve emphasis rather than peripheral mention. ACLF is defined by organ failure severity, systemic inflammation, and short-term risk, and CLIF-SOFA, CLIF-C ACLF, and CLIF-C AD are the scores that best align with those dimensions [44]. In practical terms, CLIF-C ACLF is the most useful overall prognostic score once the syndrome is established, whereas CLIF-C AD is more useful in patients with acute decompensation who are still below the ACLF threshold but remain at risk of deterioration. These scores do not eliminate clinical judgement, but they provide the clearest structured framework for daily hepatology and ICU decision-making because they track the syndrome that clinicians are actually treating rather than only the chronic severity of liver disease [4,5,44] (Table 3).

Even so, biomarkers remain adjunctive rather than definitive. C-reactive protein, lactate, leukocytosis, and serial creatinine trends remain clinically useful because they signal inflammation, tissue hypoperfusion, infection, and evolving renal injury, but they do not define ACLF by themselves. More refined biomarker work has shown that inflammatory and macrophage activation markers can improve prognostic performance when added to standard clinical scores, which supports the broader concept that ACLF severity is driven not only by organ failure but also by the intensity of systemic inflammation [52,56]. These markers therefore add biological depth to risk stratification, but they are most useful when interpreted within a CLIF-based framework rather than as alternatives to it.

That point matters because one of the most important advances in diagnosis has been conceptual rather than technological: repeated reassessment over the first 24 to 72 h. In ACLF, prognosis is dynamic. A patient whose kidney function improves after antibiotics, albumin, and source control is not biologically equivalent to one whose cerebral, respiratory, or circulatory dysfunction worsens despite apparently appropriate treatment. The course after admission may be more informative than the admission value itself. This principle has been demonstrated in machine-learning models as well as in conventional cohort analyses, where sequential reassessment at day 3, day 7, or later improves prediction compared with baseline estimates alone [4,44,100]. In other words, the most meaningful diagnostic advance in ACLF is often not a new assay, but disciplined re-evaluation of whether organ dysfunction is stabilizing, reversing, or progressing.

Risk stratification also needs to extend beyond laboratory values and organ support. Sarcopenia, frailty, and physical reserve increasingly behave as prognostic variables rather than background descriptors [77,78,79]. Two patients with similar bilirubin, INR, creatinine, and ACLF grade may differ profoundly in resilience, capacity for rehabilitation, and tolerance of prolonged critical illness. This has direct clinical relevance because advanced liver disease is not only a biochemical disorder but also a syndrome of reduced extrahepatic reserve [77,78,79,80]. More recent data on liver frailty indices in ACLF suggest that functional reserve retains prognostic importance even during hospitalization, again reminding us that trajectory is shaped by more than conventional laboratory severity alone [80].

Risk is also phenotype-dependent. Not every ACLF admission behaves the same way. In acute variceal bleeding, ACLF clearly identifies a subgroup with worse outcomes, yet the relative prognostic contributions of organ failure, baseline hepatic reserve, and procedural management can differ across cohorts [97,98]. The same is true for infection-associated ACLF, renal-predominant ACLF, and HBV-related ACLF. This is where contemporary prognostication is moving: toward models that remain anchored in organ failure severity, but are interpreted within the clinical phenotype in front of the team rather than as a single universal mortality estimate.

Artificial intelligence and machine learning fit into this evolution most usefully when they are kept close to the main clinical arc of ACLF care: dynamic risk stratification, ICU decision-making, transplant timing, and prediction of short-term outcomes. Their value is not that they replace bedside medicine, but that they may detect complex, nonlinear interactions among organ failures, inflammatory markers, treatment response, and evolving physiology that conventional models flatten or miss [101]. AI applications in ACLF have already been reviewed as a distinct field, with emerging roles in short-term mortality prediction, resource allocation, and mechanistic understanding of morbidity and mortality patterns [102]. Newer ICU-based machine-learning models in patients with ACLF and multiple organ failures have shown robust discrimination and calibration, in some cohorts outperforming conventional scores such as CLIF-C ACLF and MELD 3.0 [103]. That is potentially important not because it makes CLIF-C obsolete, but because it may help refine the difficult decisions that occur on top of CLIF-based staging, especially in critically ill patients where short-term prognosis is most uncertain.

The most convincing AI studies are also the ones that mirror what experienced clinicians already know: ACLF is a moving syndrome, and repeated reassessment improves prediction. Multicenter studies have shown that machine-learning models can improve prediction of 28-day mortality, particularly when sequential reassessment is incorporated, which is fully consistent with the broader principle that prognosis in ACLF is determined not only by where the patient starts, but by whether organ dysfunction improves or worsens after treatment begins [100]. In practical terms, this places AI in the service of the same questions that matter clinically: Is the patient stabilizing? Is ICU support changing the trajectory? Is transplant evaluation becoming more urgent? Or is short-term mortality remaining unacceptably high despite appropriate care?

This same approach is expanding into biologically enriched prediction. In HBV-related ACLF, machine-learning models built around inflammatory protein signatures have shown very strong discrimination for 90-day mortality, reinforcing the idea that inflammatory biology is not only explanatory but potentially operational in future risk stratification [104]. ML has also been used to identify distinct ACLF clusters with different trajectories, reversibility, and mortality, suggesting that hidden phenotypes may exist within what clinicians still label as one syndrome [105]. That matters because heterogeneity remains one of the central barriers in ACLF research and one of the main reasons why apparently similar patients often behave differently at the bedside.

AI may also become clinically relevant beyond prognosis alone. In principle, ML tools could help identify patients at high risk of AKI, refractory shock, or early treatment failure, allowing earlier escalation, more rational ICU triage, and timelier transplant discussions. This is no longer purely theoretical. Comparative models have already shown good performance for prediction of AKI in ACLF [106], and expert-augmented machine learning has been explored for post-transplant outcomes in ACLF, suggesting that computational approaches may help integrate clinical expertise with large-variable prediction rather than compete with it [107]. Similar work in post-transplant ACLF cohorts has shown that ML may outperform conventional models in short-term survival prediction after liver transplantation, which may become relevant for allocation strategy and peri-transplant counseling [108]. In that sense, AI in ACLF is most useful when understood as a support for management decisions that are already central to the syndrome: who to monitor more closely, who to escalate earlier, and who to discuss with transplant teams sooner.

Still, enthusiasm needs discipline. Most AI models remain dependent on retrospective data, local case mix, incomplete external validation, and uncertain real-world implementation [109,110,111,112,113,114,115,116]. Explainability, calibration, transportability across populations, and the risk of embedding bias into allocation or escalation decisions remain major concerns [102,103,104,105,109,110,111,112,113,114,115,116]. For now, the most reliable structure for everyday practice remains staged organ failure assessment with CLIF-based scoring, repeated clinical reassessment, and phenotype-specific interpretation of reversibility. AI should be viewed as an adjunct layered on top of that structure, not as a substitute for it [101].

Taken together, advances in diagnosis and risk stratification did not eliminate uncertainty in ACLF, but they made it more clinically structured. We are better at distinguishing acute decompensation from ACLF, better at identifying CLIF-SOFA, CLIF-C ACLF, and CLIF-C AD as the most useful scores for staging and short-term prognostication in everyday practice, better at recognizing that prognosis depends on inflammation, frailty, and extrahepatic reserve as much as on bilirubin or INR, and increasingly better at using computational tools to refine dynamic bedside decisions in the ICU and transplant setting rather than treating ACLF as a static event. The next step is not to move away from CLIF-based clinical reasoning, but to strengthen it with tools that reflect the syndrome more faithfully and more dynamically than liver-specific scores alone ever could.

## 7. Contemporary Management: Parallel Rather than Sequential Care

The practical management of ACLF begins with abandoning sequential thinking. These patients do not deteriorate one organ at a time, and management cannot proceed one problem at a time either. Contemporary care is therefore parallel from the outset: define and treat the precipitating event, stage organ failures using ACLF-specific scores, establish hemodynamic and volume status, support the organs already failing, anticipate those likely to fail next, and determine early whether the trajectory is moving toward recovery, transplantation, or futility [4,11,17,63,64,65,117,118,119,120,121,122]. This is not a stylistic preference. It is a consequence of the syndrome itself. Infection, renal dysfunction, encephalopathy, bleeding, alcohol-related hepatitis, and circulatory failure usually coexist and interact, and delay in one domain often erodes the gains made in another.

That initial assessment should be structured. Prognosis in the critically ill cirrhotic patient is better assessed with scores that include extrahepatic organ failure, such as CLIF-C, NACSELD, or AARC, than with MELD or MELD-Na alone, and serial recalculation is more informative than a single baseline value [4,44,117]. The same principle applies clinically. The first hours should establish not only liver-related severity but also brain failure, shock, respiratory compromise, kidney injury, bleeding risk, nutritional status, and candidacy for escalation. In this setting, West Haven criteria and Glasgow Coma Scale remain practical tools for grading severe hepatic encephalopathy, bedside echocardiography is useful in hypotension and shock, and early ICU-level monitoring is appropriate when there is severe encephalopathy, vasopressor requirement, respiratory failure, or evolving multiorgan dysfunction [4,117,118,119,120,121,122].

Infection remains the central practical target because it is both frequent and modifiable. Every hospitalized patient with ACLF, and indeed every patient with cirrhosis whose clinical status changes through new ascites, worsening encephalopathy, AKI, organ failure, or hypotension, should undergo a full infectious workup that includes diagnostic paracentesis when ascites is present, blood cultures, urinalysis and urine culture, and chest imaging [1,4,11,117,123,124]. This evaluation should be repeated when the clinical course shifts. Empirical antimicrobial therapy should begin early, guided by infection source, severity, community versus nosocomial acquisition, prior antimicrobial exposure, and local resistance ecology, with later de-escalation when microbiological data allow [4,11,117,124]. In ACLF, waiting for culture confirmation is often a mistake. Delayed or inadequate first-line antimicrobial therapy worsens prognosis, whereas prompt appropriate therapy can alter the trajectory [4,11,63,64,65,117,118,119]. At the bedside this means maintaining a low threshold not only for treating bacterial infection, but also for broadening coverage when a nosocomial course, multidrug resistance, or failure to improve after 48 h raises concern for resistant bacteria or fungal infection [4,117]. Prevention matters as well: unnecessary proton pump inhibitors and urinary catheters should be minimized because they are not benign in this population [4].

Hemodynamic management is equally practical and equally unforgiving. Every critically ill patient with cirrhosis should undergo early assessment of perfusion, intravascular volume, and cardiovascular function, because shock in ACLF is rarely explained by one mechanism alone [117,118,119,120]. Balanced crystalloids and albumin each have a role, but neither should be used reflexively. Volume resuscitation should be judicious and guided by repeated clinical and hemodynamic assessment rather than by protocolized fluid loading, particularly in patients with tense ascites, pleural effusions, latent cardiomyopathy, or respiratory compromise [4,117,124]. A mean arterial pressure around 65 mm Hg remains a reasonable initial target in septic shock, but end-organ perfusion matters more than the number itself. Norepinephrine is the preferred first-line vasopressor; vasopressin is an appropriate second-line agent when escalating norepinephrine requirements suggest refractory vasodilatory shock [4,117]. Hydrocortisone may be considered in refractory shock requiring high vasopressor doses, although recent trial data do not support routine benefit in all cirrhotic septic shock patients and reinforce that steroid use should be selective rather than automatic [4,75,117,124,125,126,127,128,129,130]. The broader practical point is that circulatory support in ACLF is not a generic ICU exercise: it is one of the few ways to buy meaningful time, but only if that time is being used to reverse the precipitant or move toward transplantation.

Renal support deserves special emphasis because kidney failure is both common and, at least in part, actionable. In cirrhosis with AKI, diuretics and nephrotoxins should be withdrawn, precipitating factors treated, and albumin challenge used where appropriate before labeling the process HRS-AKI [4,117,123,124], asalbumin leads to a faster improvement in hemodynamics and lactate clearance [131]. For patients who meet criteria for stage 2 or greater HRS-AKI and have no contraindications, vasoconstrictors plus albumin remain standard. Terlipressin is indicated in appropriately selected hospitalized patients without ACLF-3 or major cardiopulmonary disease, whereas norepinephrine is a valid alternative and may be preferable when shock coexists [4,117,123,124]. Albumin remains part of this strategy, but the era of thinking about HRS treatment as a purely renal protocol is over. In ACLF, kidney injury sits inside a wider syndrome of inflammation, vasodilation, impaired cardiac reserve, and organ hypoperfusion [4,11,58,64,123,124]. That is why treatment response must be interpreted in context. Falling creatinine is meaningful, but it does not by itself mean that ACLF has reversed. Renal replacement therapy should therefore be individualized and used most purposefully in patients who have failed pharmacotherapy and are listed or actively being considered for liver transplantation [4,117,124], as among unlisted patients, mortality rates are extremely high ineither HRS or acute tubular necrosis [132,133,134,135].

Neurologic management should also be practical and disciplined, as it impairs survival independently of failure of other organs [136,137,138,139]. Severe hepatic encephalopathy warrants ICU consideration, particularly at West Haven grades 3 or 4 or with Glasgow Coma Scale below 8 [4,117]. Empirical treatment of encephalopathy and investigation of precipitants should proceed together. Lactulose, given orally or rectally, remains first-line; polyethylene glycol is a reasonable alternative when ileus or abdominal distension make standard purgation difficult [4,117]. Patients who do not behave like prior episodes of encephalopathy, or who fail to improve despite adequate therapy, require evaluation for non-hepatic causes of altered mental status such as alcohol withdrawal, structural brain injury, drug toxicity, or sepsis-related encephalopathy [4,117]. Routine ammonia testing adds little in this setting, and routine brain imaging is unnecessary when the presentation is typical and responsive, but both have a place when the neurologic picture is atypical. If intubation is required, sedatives with short half-lives such as propofol or dexmedetomidine are preferred because prolonged oversedation quickly obscures the real neurologic trajectory [4,117].

Respiratory support has become a larger part of ACLF management than older hepatology algorithms acknowledged. Respiratory failure may reflect pneumonia, hydrothorax, tense ascites, volume overload, acute lung injury, or a mixed septic and circulatory picture [4,117,120,121,122]. Management should therefore include active search for cirrhosis-specific contributors such as hepatic hydrothorax or abdominal compartment effects from tense ascites, both of which may improve with drainage. High-flow nasal cannula is reasonable in acute hypoxemic respiratory failure with close monitoring for escalation, while invasive ventilation should use lung-protective strategies rather than “cirrhosis-specific” permissiveness: low tidal volumes, limitation of plateau pressures, and tailored PEEP, with lower PEEP in mild lung injury when venous return is precarious and higher PEEP reserved for more severe hypoxemia [4,117]. Mechanical ventilation in ACLF is not merely supportive; it changes preload, sedation needs, encephalopathy assessment, and transplantability, so it must be integrated into the overall trajectory rather than treated as an isolated organ intervention.

Bleeding and coagulation management in ACLF also require a modern frame. INR should not be used as a surrogate for bleeding risk in cirrhosis, and hemostasis in ACLF cannot be understood through conventional coagulation tests alone [4,117]. Portal hypertension remains the dominant driver of most major bleeding, particularly variceal bleeding, while the global hemostatic state is more complex and only imperfectly captured by thrombin generation assays or viscoelastic testing, neither of which has yet been fully validated for routine decision-making [4,117,140,141,142,143]. This means transfusion and anticoagulation decisions should be individualized, especially in severe thrombocytopenia, rather than guided by outdated assumptions that all cirrhotic patients are “auto-anticoagulated” or uniformly prone to procedural bleeding. Point-of-care coagulation tests like thromboelastography and Sonoclotcan better detect coagulation defects and transfusion thresholds in ACLF and ESLD, whilethese conventional tests appear to be less suitable in patients with clinical bleeding [140,141,142,143].

Nutritional care should no longer be an afterthought. Early nutrition team involvement is recommended, and objective assessment of nutritional risk at ICU admission is appropriate because ACLF patients are frequently sarcopenic, catabolic, and vulnerable to refeeding problems [77,78,79,117]. Energy targets should be modest initially and then increased as the patient stabilizes; protein restriction is not recommended, even in encephalopathy, and enteral nutrition should be favored whenever feasible [4]. The practical caveat is that enteral feeding should be held in patients requiring high-dose vasopressor support, and electrolytes should be monitored closely for refeeding syndrome. Glucose targets should remain within usual ICU ranges rather than chase “normality” at the cost of hypoglycemia [117]. In other words, supportive care in ACLF is not ancillary to definitive treatment; for many patients it is what determines whether recovery or transplantation remains biologically plausible.

All of this management remains dynamic. ACLF-specific scores should be recalculated serially, but more importantly the bedside team should ask the same questions every day: Is the precipitating event controlled? Are organ failures stabilizing, reversing, or accumulating? Is the patient becoming more transplantable, or less? This repeated reassessment is what turns parallel care into intelligent care [4,44,117,118,119,120,121,122]. It also helps avoid two common errors: persisting with maximal organ support after reversibility has disappeared, or delaying transplant evaluation until organ failure has advanced beyond rescue. Early transplant assessment should therefore occur alongside, not after, aggressive management [4,117,118,119,120,121,122,144,145]. Expedited transplantation may be justified in selected patients, but decisions about futility should rest on reversibility, candidacy, available resources, and the realistic likelihood that support is bridging to something meaningful.

Finally, practical care in ACLF also includes palliative care, not as surrender but as good medicine. A palliative care consultation should be considered in critically ill patients with cirrhosis and ACLF to clarify prognosis, define goals of care, and support communication, while disease-directed treatment and transplantation evaluation continue in parallel [4,14,15,117]. Primary palliative care can and should be delivered by the treating team, with specialist involvement when available. This parallel approach matters because the same patient may still be a transplant candidate and also be suffering from dyspnea, fear, family uncertainty, and the burdens of intensive treatment. Good ACLF care therefore requires practical simultaneity at every level: prompt antimicrobials, disciplined hemodynamics, rational renal support, active neurologic and respiratory management, nutrition, serial prognostication, early transplant thinking, and early communication about goals and limits. That is what parallel care means in real life.

## 8. HRS-AKI: Complex and Treatable Component of ACLF

HRS-AKI is one of the clearest examples of how ACLF care has become more structured and more actionable. It is no longer acceptable to treat it as a vague late-stage renal event in advanced cirrhosis. Current practice requires a positive diagnostic framework and not merely therapeutic pessimism. In practical terms, HRS-AKI should be suspected in a patient with cirrhosis and AKI that persists despite withdrawal of diuretics, treatment of precipitating factors, and volume expansion with albumin, once shock, persistent hypovolemia, structural kidney disease, and substantial nephrotoxin exposure have been reasonably excluded [4,120,124]. This distinction matters because the diagnosis carries immediate therapeutic consequences. It identifies a form of kidney injury driven predominantly by severe circulatory dysfunction and renal vasoconstriction within the broader inflammatory syndrome of ACLF, rather than by intrinsic parenchymal damage alone [4,58,124].

That diagnostic discipline is particularly important in ACLF, where AKI is common but heterogeneous. Not every creatinine rise in a patient with cirrhosis is HRS-AKI, and the temptation to use vasoconstrictors empirically in any severe AKI should be resisted. Real-world data confirm that terlipressin is frequently used outside its primary indication and that response is substantially lower in acute tubular necrosis than in true HRS-AKI, while respiratory complications remain clinically relevant [146]. The diagnosis therefore still begins at the bedside: establish the temporal relationship to infection, bleeding, or overdiuresis; review medications; assess urine output and sediment when helpful; evaluate for shock and sepsis; and determine whether the kidney injury is occurring in a vasodilated, underfilled circulation rather than in a patient with established intrinsic renal injury. In ACLF, this is rarely a purely renal exercise. It is part of defining whether renal dysfunction represents a potentially reversible hemodynamic complication or one more expression of irreversible multiorgan failure.

The therapeutic rationale follows directly from that physiology. Both terlipressin and norepinephrine work by increasing mean arterial pressure and counteracting the profound splanchnic and systemic vasodilation of advanced cirrhosis. By restoring effective arterial blood volume, reducing pathological vasodilation, and improving renal perfusion pressure, they reduce the intense renal vasoconstriction that characterizes HRS-AKI [4,117,124,147,148,149]. In other words, these drugs do not “treat the kidney” directly; they treat the circulatory disorder that is starving the kidney. Recent post hoc analysis of clinical trial data has reinforced this pharmacodynamic model, showing that terlipressin produces an early and sustained increase in mean arterial pressure and that rises in MAP are directly associated with a greater likelihood of HRS-AKI reversal [150]. This is why bedside hemodynamic reassessment remains essential during therapy. If MAP is not improving, renal recovery becomes less likely, regardless of what has been prescribed.

Among available therapies, terlipressin has the strongest body of evidence and remains the preferred vasoconstrictor where available and appropriate [4,117,123,124,147,148,149,151,152]. The CONFIRM trial showed that terlipressin plus albumin improves HRS reversal compared with placebo plus albumin, confirming that this form of AKI is, at least in part, modifiable when recognized early and treated within a protocolized framework [123]. Subsequent meta-analyses have been consistent on this point: terlipressin improves HRS reversal, even though a clear mortality benefit has been harder to demonstrate across trials [147,152]. That apparent paradox is clinically familiar in ACLF. Renal reversal may still be worthwhile because it can reduce the need for renal replacement therapy, improve hemodynamic stability, preserve transplant candidacy, and create time for recovery from the precipitating insult, even when it does not independently determine long-term survival [4,123,124].

Norepinephrine remains a valid and important alternative, especially in ICU settings. Randomized studies in classic type 1 HRS have generally shown norepinephrine to be broadly comparable to terlipressin for HRS reversal, with no clear consistent difference in short-term survival [153,154,155,156]. This is why current guidance accepts norepinephrine as an alternative vasoconstrictor and, in some ICU patients with concurrent shock, a practical first choice [117,124]. The nuance is that ACLF may not behave exactly like less complex decompensated cirrhosis. In a randomized trial focused specifically on ACLF patients with HRS-AKI, terlipressin achieved earlier and more frequent response than norepinephrine and was associated with lower renal replacement therapy use and better 28-day survival, although at the cost of more reversible adverse events [157]. This does not invalidate norepinephrine, but it does suggest that equivalence between the two agents should not be assumed in every ACLF phenotype. The choice therefore remains contextual: terlipressin when available and tolerated in carefully selected patients; norepinephrine when ICU-level titration is needed, when shock predominates, or when terlipressin is contraindicated or poorly tolerated [117,124,153,154,155,156].

How terlipressin is given also matters. Continuous infusion appears better tolerated than intermittent bolus administration and may achieve similar efficacy at lower effective doses [158]. This has practical relevance because adverse events are one of the main limitations of therapy. More recent transplant-center data using continuous terlipressin infusion showed favorable response rates and acceptable safety in selected transplant-eligible patients, again supporting the idea that technique and patient selection influence outcome as much as the drug itself [159]. There is also early evidence that in patients showing early non-response to terlipressin, the addition of norepinephrine may increase the probability of HRS resolution with fewer adverse effects, although this remains a small-study signal rather than standard care [155]. For now, it is best viewed as an interesting rescue strategy in specialized settings, not as a routine starting regimen.

Safety, however, is not a minor caveat. It is central to the use of terlipressin in ACLF. Respiratory failure and hypoxemia are clinically significant adverse events, particularly in patients with advanced ACLF, pneumonia, baseline hypoxemia, cardiopulmonary fragility, or limited reserve to tolerate further shifts in preload and afterload [123,124], as respiratory failure is especially concerning in patients with grade 3 ACLF and in those with low baseline oxygen saturation or more severe circulatory compromise [146]. This is why terlipressin should not be treated as a creatinine algorithm detached from the rest of the patient. Oxygenation, chest imaging, fluid balance, cardiopulmonary status, and ACLF severity all matter before and during treatment. In practice, one should think of terlipressin as a targeted hemodynamic intervention, not as a benign renal drug.

Protocolization itself also seems to matter. Real-world data from a structured evidence-based protocol for diagnosis and treatment of HRS showed an independent association with lower mortality, suggesting that standardization is part of the therapy and not just a quality-improvement footnote [60]. That is believable clinically. HRS-AKI responds best when diagnosis is made early, albumin is used thoughtfully, infection is controlled, nephrotoxins are removed, hemodynamics are reassessed frequently, and escalation decisions are made without delay. In ACLF, inconsistency is often lethal not because it changes one laboratory value, but because it wastes the narrow window in which renal dysfunction may still be reversible.

Renal replacement therapy sits further downstream and should be framed honestly. It can correct acidosis, potassium, and volume excess, but it is not a treatment for HRS-AKI itself and rarely changes the biology of ACLF unless it is buying time for liver transplantation or recovery from a reversible precipitant [117,132,133,134,135]. Outcomes after initiation of renal replacement therapy in cirrhosis remain poor, particularly in transplant-ineligible patients and in those with sustained multiorgan failure [132,134,135]. Management of acute RRT in critically ill cirrhotic patients therefore requires individualized decisions about indication, timing, modality, and above all purpose [133]. Used well, it is a bridge. Used indiscriminately, it may simply prolong a dying process without changing its direction.

For that reason, HRS-AKI should be understood as one of the most treatable components of ACLF, but never as a self-contained disease. A fall in creatinine is valuable, yet it is not equivalent to reversal of ACLF. It may preserve transplantability, reduce the need for RRT, and restore some physiologic reserve, but outcome may still be determined by persistent infection, worsening encephalopathy, respiratory failure, or progressive circulatory collapse [4,44,124]. The most honest way to frame therapy is therefore as a bridge strategy: bridge to recovery in some patients, bridge to transplantation in others, and bridge to clearer decision-making when biology does not improve. In ACLF, that is often what good renal therapy really means.

## 9. Intensive Care, Extracorporeal Support, and the Limits of Rescue Therapy

Intensive care is now central to the management of severe ACLF, but it should be understood for what it is: a setting in which time can be won, not a therapy that by itself reverses the syndrome. Mechanical ventilation, vasopressors, renal replacement therapy, correction of metabolic derangements, and invasive monitoring can stabilize physiology long enough to control a precipitating event, allow organ recovery, or reach liver transplantation, but they do not directly extinguish the inflammatory and circulatory biology driving ACLF [145,160,161,162,163,164,165,166,167,168,169]. This distinction is practical rather than philosophical. ACLF is a multisystem syndrome involving hepatic dysfunction, portal circulatory derangement, immune dysregulation, cerebral dysfunction, renal failure, and cardiovascular instability. ICU care is therefore not an optional add-on for a few extreme cases, but often the only environment in which organ support, close reassessment, and transplant-oriented decision-making can occur at the speed the syndrome demands [145,168,169,170].

That said, the role of ICU care is not open-ended maximalism. In most cases, it is more useful to think in terms of an initial trial of intensive care over the first 48–72 h, with explicit goals and equally explicit reassessment. The purpose of that trial is not merely to demonstrate that organ support can be delivered, but to determine whether support is being converted into something biologically meaningful: control of infection or bleeding, improving hemodynamics, falling lactate, stabilization or reversal of organ failures, or movement toward transplantation [4,117,171,172]. This is where ACLF-specific prognostic tools become clinically useful. Conventional cirrhosis scores are often inadequate in this setting, whereas CLIF-based models better capture the short-term risk associated with multiorgan dysfunction [4,5,44,117]. Serial reassessment matters even more than admission severity. In ICU patients with ACLF, a persistently very high CLIF-C ACLF score after 48 h may identify a threshold beyond which continued maximal support is unlikely to change outcome, particularly in those who are not transplant candidates [171,172]. The implication is not therapeutic nihilism, but disciplined reassessment.

This is particularly important because ACLF can look deceptively “supportable” for a period of time. Vasopressors can restore arterial pressure, ventilation can normalize oxygenation, and renal replacement therapy can correct potassium and acid–base status, yet the patient may still be biologically deteriorating. The bedside question is therefore not whether support is technically feasible, but whether it is changing trajectory [4,44,161,162,163,164,165,166,167,168,169]. Progressive respiratory failure despite appropriate ventilatory support, refractory shock, worsening encephalopathy, new infections, or accumulation of organ failures despite correction of the original precipitant should trigger reappraisal rather than automatic escalation. In practice, that is one of the most important functions of ICU care in ACLF: not only support, but clarification of reversibility.

For patients already listed for liver transplantation, or clearly suitable for expedited listing, the threshold for aggressive ICU support should be correspondingly higher. In that group, organ support is often justified precisely because it can bridge to a therapy that changes prognosis. Recent data have reinforced this point. Organized early access to transplantation in critically ill ACLF patients can produce meaningful survival benefit, and longer-term outcomes after liver transplantation in ACLF grade 3 are better than older assumptions suggested [58,59]. Indeed, long-term follow-up now shows that 5- and 10-year patient and graft survival after transplantation for ACLF-3 can approach those of less severe ACLF groups when selection is careful [143]. This matters greatly for ICU decision-making. In a transplant-eligible patient, particularly one already listed or under urgent evaluation, the presence of ACLF alone should not be used to justify therapeutic retreat. Post-transplant mortality does not increase so dramatically as to make aggressive pre-transplant support irrational [144,145,169]. On the contrary, in such patients, it is often reasonable to invest heavily in organ support, provided that trajectory remains compatible with transplantation.

The opposite problem arises in patients who are not transplant candidates, or whose biology is evolving away from candidacy despite full support. Here, the danger is not undertreatment, but prolonged technologically sophisticated treatment without a coherent endpoint. This is where ICU care reaches its ethical and clinical limits. Mechanical ventilation may be entirely appropriate as a bridge in potentially reversible ACLF, but much less so when uncontrolled sepsis, profound neurologic injury, or irreversible multiorgan failure make recovery implausible. Renal replacement therapy may correct chemistry, but it does not reverse ACLF by itself. In these circumstances, palliative care is not a failure of intensive care; it is part of good intensive care. The 48–72 h ICU trial is helpful precisely because it allows teams to distinguish early between support that is buying time for recovery or transplantation and support that is only prolonging dying [4,14,15,117]. In patients who are not transplantable and who continue to worsen despite appropriate therapy, a shift toward comfort-focused and proportionate care is often the more honest and humane decision.

Extracorporeal liver support remains attractive for the same reason ICU support is attractive: it promises to buy time while the precipitant is treated or transplantation is arranged. Yet here too the field remains ahead of the evidence. Earlier reviews already recognized extracorporeal liver assist devices as conceptually important but clinically unproven [167]. More recent work has not fundamentally changed that conclusion. The DIALIVE trial is notable because it showed biological and score-based improvement, including faster ACLF resolution and reduced inflammatory and endothelial dysfunction markers, but it was small and not powered to demonstrate a survival benefit [173]. Plasma exchange is similarly promising, with systematic reviews and meta-analyses suggesting possible short-term survival benefit in ACLF, particularly as a bridge in selected patients, but randomized evidence remains insufficient to establish it as standard therapy across ACLF phenotypes [170,174,175,176]. The practical conclusion is therefore cautious: extracorporeal support may have a role as a bridge in specialized centers, especially in transplant-oriented strategies, but should not be mistaken for a validated disease-modifying treatment for ACLF in general practice.

What ICU care does offer reliably is structure: continuous observation, protocolized organ support, repeated scoring, and multidisciplinary decision-making at the speed ACLF requires. That makes it the right place not only for escalation, but also for determining when escalation has ceased to be proportionate. In this sense, the ICU is not simply where ACLF is treated; it is where its reversibility is tested. Some patients will improve quickly when infection is controlled and perfusion restored. Others will stabilize enough to reach transplantation. Others will demonstrate, despite technically correct care, that rescue is failing. The task is to recognize which of these paths the patient is on before time is lost.

Intensive care therefore has a major role in modern ACLF, but it is a conditional role. It is most valuable when used as a bridge with a defined purpose, especially in transplant-eligible patients in whom aggressive support may convert into long-term survival [144,145]. It is least useful when pursued indefinitely in patients whose biology is no longer salvageable. Between those extremes lies the real work of the ICU team: to support early, reassess honestly, and know when to continue fighting and when palliation has become the better form of care.

## 10. Liver Transplantation: Timing Matters as Much as Candidacy

Liver transplantation remains the only truly definitive treatment for many patients with ACLF and for a substantial proportion of those who have progressed into ESLD. That distinction has practical consequences at the bedside. Transplant thinking should begin early, often at the same time as resuscitation and organ support rather than after “failure” of intensive care [4,177,178,179,180].

What has changed in recent years is not that principle, but the threshold for applying it. The field has moved away from the older reflex of treating very severe ACLF as a near-automatic contraindication and toward a more evidence-based position: selected patients with advanced multiorgan failure, including ACLF grade 3, can achieve acceptable short- and long-term outcomes after transplantation when selection is careful and timing is right [144,145,168,177,178,179]. This is an important correction in perspective. ACLF undoubtedly increases urgency, complexity, and perioperative risk, but it does not abolish transplant utility. In fact, contemporary data increasingly suggest that ACLF itself does not impair post-transplant survival nearly as much as once feared; what matters more is whether the patient remains biologically salvageable at the time of transplantation [145,168,177,178,179,180].

In ACLF, waiting for perfect stabilization is frequently the wrong instinct, because some organ failures will not fully reverse before transplantation and do not need to. The more relevant question is whether the trajectory is interpretable and whether support is buying meaningful time. A patient with improving infection control, stabilizing hemodynamics, falling vasopressor requirement, recovering renal function, or at least non-progressive organ failure may still be moving toward a transplant opportunity even while critically ill. By contrast, persistent uncontrolled sepsis, worsening respiratory failure, severe frailty, or irreversible neurologic injury usually indicate not merely severity, but diminishing plausibility of benefit [4,168,177,178,179,180]. In this sense, candidacy in ACLF is dynamic. It is less a fixed label than a judgment repeatedly refined over days.

This is why timing matters as much as candidacy. The transplant window in ACLF is narrow and unstable. List too late, and the patient may move beyond rescue. List too early, without understanding whether the course is potentially reversible or simply escalating toward futility, and the team risks transplanting into a biologically incoherent situation. Recent reviews have framed this tension clearly: the central challenge is no longer whether transplantation can work in ACLF, but how to identify the moment at which short-term mortality without transplant is high while post-transplant recovery remains realistic [180]. The prospective national prioritization data and long-term follow-up studies point in the same direction. Transplantation in critically ill ACLF patients can be justified and effective, but only when embedded in systems able to recognize urgency early, reassess frequently, and integrate ICU and transplant decisions closely [144,145].

The newer long-term outcome data are especially important because they challenge one of the most persistent objections to transplanting severe ACLF. In carefully selected patients with ACLF grade 3, 5- and 10-year patient and graft survival have been shown to be comparable to those of matched controls with less severe or no ACLF [145]. That finding should not be overstated, but it is hard to ignore. It means that, from a utility perspective, transplantation in ACLF-3 can still be justified and that the old assumption of prohibitive long-term futility is no longer tenable in experienced centers [145]. The message is not that every patient with ACLF-3 should be transplanted, but that severity grade alone is an inadequate reason to deny transplantation. Comorbidity burden, infection control, neurologic status, cardiopulmonary reserve, and the reversibility of organ failures are likely more informative than ACLF grade in isolation [177,178,179,180,181].

At the same time, transplantation should not be pursued in a vacuum. The ICU course before transplantation matters because it helps distinguish salvageable biology from non-beneficial escalation. Data validating CLIF-C ACLF thresholds after 48 h of intensive care are relevant here, not because they provide an absolute futility rule, but because they reinforce a principle that transplant teams already recognize clinically: repeated assessment is more informative than admission severity alone [169]. A persistently extreme CLIF-C ACLF score after appropriate ICU support may identify patients in whom ongoing support is no longer moving toward transplantability [169]. That does not mean the score should replace judgment, but it can help structure difficult conversations about whether the patient is improving, stabilizing sufficiently to proceed, or drifting beyond a meaningful transplant window. In other words, ACLF transplantation is not a race to operate on the sickest possible patient; it is an effort to intervene before the biology becomes irrecoverable.

Center experience matters enormously in this process. Successful transplantation in ACLF is not only a surgical question. It depends on ICU-transplant integration, rapid multidisciplinary review, access to organs, institutional willingness to evaluate very sick candidates quickly, and allocation systems capable of recognizing short-term risk rather than relying only on chronic disease models [4,178,179,180]. It also depends on understanding what pre-transplant optimization can and cannot achieve. Mechanical ventilation, vasopressors, renal replacement therapy, and other forms of organ support may be entirely appropriate when they are building toward transplantation, but much less so when they are only prolonging physiologic collapse without a viable path forward. Modern prognostic tools, including machine learning approaches, may eventually refine these decisions further, but at present the essential ingredients remain careful clinical judgment, repeated reassessment, and close collaboration between hepatology, intensive care, and transplant teams [109,110].

Just as importantly, transplantation should not be presented as an alternative to palliative care. In ACLF, palliative care can and should be integrated alongside active transplant evaluation, because good transplant medicine also requires symptom control, clear communication, support through uncertainty, and careful shared decision-making even when transplantation remains the intended goal [14,15]. Families are usually able to understand this when it is explained clearly: that the team is pursuing recovery or transplantation actively, while also preparing honestly for the possibility that the patient may not survive or may become ineligible despite maximal support. This parallel approach often improves rather than weakens decision-making, because it keeps the focus not only on what can technically be done, but on what remains proportionate and in the patient’s interests. In ACLF, the best transplant care is rarely about a simple yes-or-no judgment. It is about recognizing the right patient, at the right time, before that opportunity closes.

## 11. Integrated Palliative Care in ESLD and ACLF

The growing place of palliative care in hepatology reflects an overdue correction rather than a new luxury. For too long, patients with advanced liver disease were managed as if the only meaningful clinical question was whether they might still reach transplantation, while the daily burden of symptoms, recurrent hospitalization, frailty, encephalopathy, caregiver exhaustion, and uncertainty remained under-addressed. The AASLD guidance on palliative care in decompensated cirrhosis has helped make that blind spot much harder to ignore, and rightly so [15]. This is particularly relevant in ESLD, where transplant candidacy may change abruptly, where reversibility is often uncertain, and where patients can move back and forth between partial recovery and clear decline in ways that are clinically and emotionally disorienting for both families and clinicians [15,16,182,183,184,185,186,187,188,189].

Palliative care in this setting should be defined clearly for readers: it is not synonymous with end-of-life care, withdrawal of treatment, or transplant ineligibility. In advanced cirrhosis and ACLF, it is better understood as an added layer of care that runs alongside hepatology, intensive care, and transplant medicine rather than after them [182,183,184,185,186,187,188,189]. Its purpose is to reduce suffering, improve communication, support complex decision-making, and align treatment with the patient’s goals while disease-directed management continues. In practice, this includes management of pain, dyspnea, pruritus, nausea, sleep disturbance, anxiety, and depression; support for surrogate decision-making; advance care planning; and structured discussions about prognosis, readmission, dialysis, ICU therapies, and what burdens a patient would or would not accept in the hope of recovery or transplantation [15,16,184,185,186,187,188]. This is especially important not only at the very end of life, but also during the long unstable phase in which recurrent encephalopathy, refractory ascites, repeated infection, sarcopenia, and progressive functional decline begin to dominate daily life even while transplant remains a live possibility [1,15,16,182,183,184,185].

This matters because ESLD is not defined only by laboratory severity or Child-Pugh class. It is also defined by symptom burden, physical reserve, social dependence, and the cumulative consequences of repeated decompensation. A patient with severe sarcopenia, poor mobility, recurrent admissions, and limited physiologic resilience may be suffering profoundly long before the final admission declares itself [77,78,79]. In this sense, integrated palliative care belongs naturally in the same conversation as ACLF. Both are concerned with thresholds of reversibility. Both require clinicians to judge whether biology still appears salvageable, whether the burdens of treatment remain proportionate, and whether the likely outcome justifies the intensity of intervention being proposed [4,15,16]. The difference is that palliative care approaches these questions explicitly and systematically instead of allowing them to emerge late, implicitly, or only after momentum has already carried care into a burdensome direction.

For that reason, palliative care should be implemented early in recognizable clinical situations rather than reserved for the final admission. These include recurrent decompensation without durable recovery, persistent high symptom burden, progressive frailty or sarcopenia, uncertain transplant candidacy, repeated ICU admissions, or increasing dependence on invasive support without clear improvement [15,16,184,185,186,187,188]. In ACLF specifically, a persistently poor trajectory after the first 48–72 h of intensive care should prompt more formal goals-of-care reassessment, not because prognostic scores replace judgment, but because they can help structure it. In this regard, a CLIF-C ACLF score above 70 after 48 h of ICU support has been associated with a threshold at which ongoing intensive care may become non-beneficial in non-transplant candidates, making palliative involvement particularly important [169,190]. This threshold should not be treated as a mechanical stopping rule, but it is clinically useful as a signal that the burden of treatment, the likelihood of recovery, and the coherence of continued escalation need to be re-examined openly.

Importantly, palliative care should not be framed as the opposite of transplantation. That misconception remains one of the main barriers to its integration in ESLD [182,183,184,185,186,187,188]. Patients awaiting transplant often have some of the greatest unmet palliative needs precisely because they live in a space of uncertainty: sick enough to suffer substantially, but not necessarily beyond rescue [184,186]. The same is true in ACLF, where ICU care may function as a bridge either to recovery, transplantation, or a clearer recognition that rescue has reached its limit [15,16,169,190]. In that setting, palliative care does not weaken disease-directed treatment. It improves communication, clarifies acceptable trade-offs, aligns interventions with patient values, supports families through uncertainty, and reduces the risk that technically possible care becomes biologically or ethically incoherent [182,183,184,185,186,187,188]. Good transplant care and good palliative care are therefore not competing models, but complementary disciplines.

**Figure 3:**
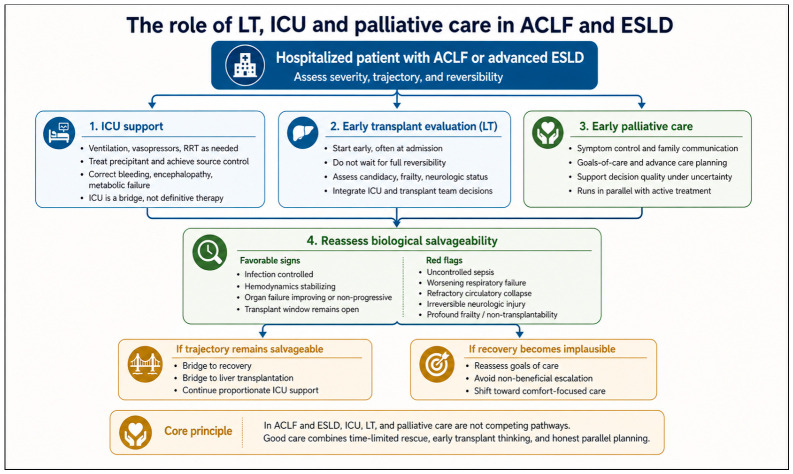
Intensive Care, Liver Transplantation and Palliative Care for ACLF and ESLD patients.

The literature has also been consistent on another point: palliative care in ESLD remains underused and, when finally introduced, is often introduced too late [182,183,184,185]. In many cohorts, referral occurs only after transplant denial, terminal decline, or a final hospitalization, by which point the opportunity for advance care planning and symptom-directed support has already narrowed considerably [184,186]. This late use encourages exactly the pattern hepatology should be trying to avoid: aggressive interventions continuing until hours before death, with transitions to comfort-focused care occurring abruptly instead of thoughtfully [182]. Earlier integration, by contrast, is associated with clearer goals of care, better code-status documentation, more healthcare proxy designation, fewer repeated admissions, and more patient-centered care planning [183]. The practical lesson is straightforward: palliative care works best when it is integrated before the final crisis, not merely activated by it (Figure 3).

Ultimately, the clinician’s duty in ESLD and ACLF is dual. One task is to recognize salvageable biology early and pursue it decisively, whether through precipitant control, organ support, or timely transplantation. The other is to recognize when recovery is no longer plausible and to prevent burdensome, non-beneficial treatment from becoming the final chapter of care [15,16,184]. Good hepatology requires both instincts. Without the first, patients lose opportunities for rescue. Without the second, they lose the chance for care that is honest, proportionate, and humane. In that sense, the work is not unlike the ethic Camus gave to physicians through Dr. Rieux in the novel The Plague: not the promise of victory over suffering, but the refusal to abandon the person in front of us [191]. In advanced liver disease, that means resisting two equal errors—nihilism when there is still a path to recovery, and false heroism when treatment no longer serves the patient. The task is not to defeat mortality at any cost, but to remain lucid, compassionate, and useful at the bedside, especially when certainty is gone.

## 12. Beyond the Tipping Point: Future Directions and Concluding Perspectives

The next phase of ACLF care will likely be defined less by a single breakthrough than by convergence. Earlier identification of patients at risk, better discrimination between reversible and irreversible trajectories, and development of treatments that modify the inflammatory, circulatory, and metabolic cascade rather than merely sustaining failing organs remain the central priorities [4,18,64]. Biomarker platforms and machine-learning approaches are increasingly attractive in this space because ACLF is a multidimensional syndrome in which prognosis emerges from interactions among inflammation, organ dysfunction, frailty, and precipitating events rather than from any single variable alone [101,102,103,104,105,106,107,108,109,110,111,112,113,114,115,116]. Even so, the benchmark they will have to exceed is not abstraction but disciplined clinical medicine: repeated bedside reassessment, interpretation of trajectory, and recognition that the same admission value can carry very different meaning depending on context.

One important direction is a more granular biology of risk. Recent work suggests that inflammatory biomarkers may help identify cirrhotic patients with AKI who are at particularly high risk of progression, dialysis requirement, in-hospital complications, and death, supporting the idea that inflammation is not only a mechanistic explanation but also a potential stratification tool [192]. In parallel, event-specific prognostication is becoming more refined. In acute variceal bleeding, newer scores such as ABC may assist with prediction of in-hospital mortality and early treatment failure in selected settings [193], while CLIF-C AD appears useful for capturing post-bleed vulnerability beyond the initial admission even in patients who do not meet ACLF criteria at presentation [194]. The broader implication is that future prognostic models will likely become both more phenotype-specific and more temporally dynamic.

Just as important is the maturation of systems of care. ACLF is unlikely ever to be managed well if overtly compartmentalized. Outcomes depend not only on what therapies are available, but also on how quickly hepatology, critical care, infectious diseases, nephrology, interventional radiology, transplant teams, nutrition support, and palliative care can function as a coherent unit [4,11,144]. In that sense, the “parallel care” model discussed earlier should be seen not merely as a bedside tactic but as an organizational standard. Future gains may come as much from shortening delays, standardizing escalation pathways, and integrating reassessment across teams as from any individual drug or device. This remains especially relevant because ACLF continues to demand a mode of care that is dynamic rather than sequential, and because the practical value of ICU support, organ support, and transplant referral still depends on how early they are integrated into a coherent plan rather than how late they are deployed.

That same systems perspective extends upstream. Contemporary portal hypertension guidance increasingly frames prevention of decompensation as a structured clinical enterprise rather than a loose collection of outpatient habits [195]. Risk stratification, bleeding prevention, early recognition of renal dysfunction, nutritional optimization, frailty assessment, and better management of cardiopulmonary comorbidity all influence whether a patient ever reaches ACLF. Likewise, allocation models continue to evolve, but recent data on MELD 3.0 remind us that even improved scoring systems do not fully capture short-term risk in advanced cirrhosis across different populations [196]. Future progress will therefore depend not only on rescuing established ACLF, but also on intervening earlier in the trajectory that leads to it.

At the same time, the field is being forced to confront its therapeutic limits more honestly. Extracorporeal liver support, immune modulation, plasma exchange, microbiome-directed strategies, and other experimental approaches remain biologically plausible but incompletely validated [4,18,167]. The most promising studies have shown signals of physiologic or biochemical benefit, but robust and reproducible disease modification has not yet been established. This has two implications. First, liver transplantation remains the definitive therapy for many patients with severe ACLF, and future work must continue to refine how urgency, reversibility, and expected post-transplant benefit are integrated into selection and prioritization [144,145]. Second, novel therapies will likely be most useful not as stand-alone solutions, but as bridges that alter trajectory enough to permit recovery or transplantation in carefully selected patients.

There is also growing interest in interventions that sit between prevention and rescue. Microbiome-directed therapies, including faecal microbiota transplantation, have generated attention because they target one of the central biological interfaces of ACLF: gut permeability, bacterial translocation, and systemic inflammation [197]. Likewise, portal decompressive strategies such as TIPS continue to be revisited in advanced populations once considered too unstable to benefit, especially where renal dysfunction and refractory ascites are prominent [198,199,200]. These approaches remain selective rather than universal, but they illustrate an important conceptual shift: the field is moving away from seeing ACLF only as a syndrome to be supported, and toward identifying points at which its pathophysiology might be interrupted.

Perhaps the most consequential shift, however, remains upstream. The concept of recompensation has changed how the natural history of cirrhosis is understood, because it makes clear that deterioration is not always unidirectional and that prevention of further decompensation is itself a form of ACLF prevention [2,21,22,23]. Viral suppression, sustained alcohol abstinence, portal pressure reduction, nutritional support, sarcopenia mitigation, infection prevention, and early treatment of renal dysfunction remain the interventions with the greatest real-world potential to reduce ACLF burden [2,21,22,23,77,78,79]. Newer multicentre data on sarcopenia strengthen this point, showing that muscle loss is not simply an epiphenomenon of advanced disease but an independent marker of progression, hospitalization, and mortality in decompensated cirrhosis [79]. If ACLF often declares itself in the ICU, its roots usually begin much earlier.

Future work will also need to connect systemic inflammation more directly to modifiable endothelial and metabolic dysfunction. Recent data suggest that albumin may do more than expand plasma volume: it may attenuate inflammation and oxidative stress in decompensated cirrhosis, reduce subsequent decompensation risk, and restore endothelial mitochondrial morphology toward a healthier phenotype, supporting the idea that vascular dysfunction is not merely secondary but part of the biological substrate through which decompensation progresses toward ACLF [201,202]. In parallel, experimental work on macrophage polarization and inflammatory amplification continues to identify potentially relevant molecular pathways, including MMP8/PPAR-γ signaling, that may eventually help distinguish biomarkers of severity from true therapeutic targets [203]. These lines of work are conceptually important because they move the field closer to therapies aimed at interrupting the mechanisms that sustain organ failure rather than only reacting once it is fully established.

Another likely direction is a more refined classification of ACLF heterogeneity across regions and clinical settings. Prospective data suggest that organ-failure based phenotyping early after onset may help reconcile some of the long-standing tension between Eastern and Western definitions, while still recognizing that liver-predominant and extrahepatic organ failure-predominant presentations do not carry the same short-term risk [204]. At the same time, prognostic assessment before transplantation is increasingly shaped not only by physiology but also by bias, context, and resource environment, particularly in alcohol-associated liver disease, where evaluator factors may still influence how transplantability is judged [205]. This matters because the future of ACLF care will depend not only on better scores, but also on better fairness and consistency in how those scores are interpreted. The same applies to transplantation strategy itself. Recent living-donor experience reminds us that timely transplantation can expand rescue options in donor-limited settings, although outcomes still worsen with higher ACLF grade and extrahepatic vulnerability, particularly chronic kidney disease [145,203,205,206,207,208]. Together, these studies reinforce that ACLF should not be approached as a single fixed syndrome, but as a dynamic spectrum in which biology, organ failure pattern, reserve, and access to timely transplantation all shape outcome.

For that reason, the future of ACLF care will depend on whether the field can connect these levels of thinking: molecular signals, bedside trajectory, multidisciplinary organization, and earlier prevention. The most useful advances may not be those that promise dramatic reversal in already irreversible disease, but those that allow clinicians to recognize dangerous trajectories sooner, direct intensive resources more intelligently, and intervene before multiorgan failure becomes self-sustaining. ACLF has emerged as one of the clearest places where modern hepatology, critical care, transplantation, and palliative medicine intersect. The field now knows far more clearly what the syndrome is, where its lethality comes from, and where current therapy succeeds or fails. The next challenge is to turn that knowledge into timelier, more selective, and more biologically coherent care.

## 13. Conclusions

**Figure 4:**
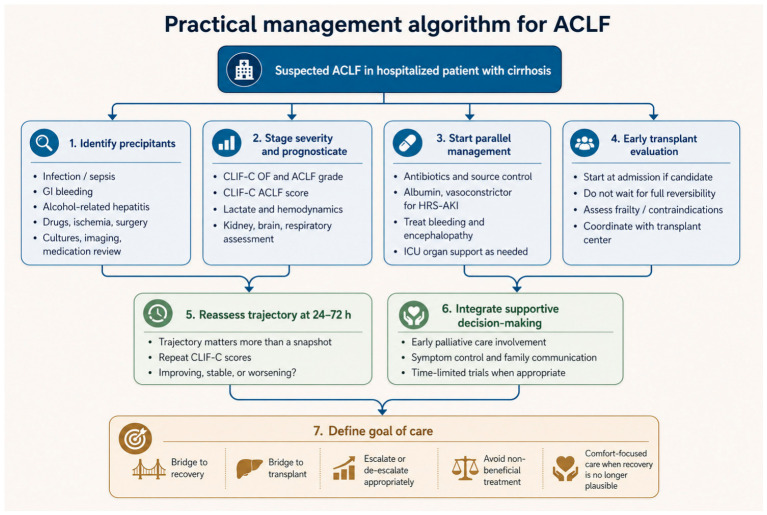
Practical management algorithm for ACLF.

Beyond the tipping point, the main lesson to be taken from this review is that ACLF demands a model of care that is dynamic, integrated, and clinically disciplined (Figure 4) (Table 4). The syndrome sits at the intersection of hepatology, intensive care, transplantation, and palliative medicine. The best outcomes are therefore unlikely to come from isolated therapeutic advances alone, but from combining early recognition, structured prognostication, prompt precipitant control, high-quality ICU support, timely transplant thinking, and honest communication about goals of care. The modern challenge in ACLF is no longer simply to recognize that these patients are critically ill, because the progress of the past decade has not eliminated the lethality of ACLF, but it has made these decisions more informed, more structured, and—if approached well—*more humane*.

## Figures and Tables

**Table 1:** Comparison of leading ACLF definitions and staging traditions.

Framework	Target Population	Formal Definition/Entry Criteria	Main Diagnostic Emphasis	Prognostic Horizon	Strengths	Limitations	Practical Implication
EASL-CLIF	Hospitalized patients with acute decompensation of cirrhosis	Acute decompensation plus organ failure(s) defined by the CLIF-C OFsystem; graded ACLF 1–3 according to number/type of organ failures and associated mortality	Extrahepatic and hepatic organ failure, severity staging, short-term mortality	Primarily 28- and 90-day mortality, CLIF-C scores 30-, 90-, 180- and 365-day mortality	Most clinically comprehensive for hospitalized cirrhotic patients; best validated in multiorgan dysfunction; strongest framework for severity staging and transplant-oriented decision-making	More complex than binary bedside systems; requires formal organ failure assessment	Best suited for bedside severity stratification, ICU-level assessment, and transplant-oriented hepatology
APASL/Kyoto/AARC tradition	Patients with chronic liver disease or cirrhosiswith an acute hepatic insult, especially in Asian practice	Acute hepatic insult leading to jaundice and coagulopathy, followed within weeks by ascites and/or encephalopathy; severity often refined with AARCscoring	Hepatic insult and liver failureat presentation, with later incorporation of systemic consequences	Primarily 28-day and short-term mortality	Easy to apply; captures hepatic-triggered ACLF well; highly relevant in HBV- and alcohol-predominant populations; robust within APASL cohorts	Less focused on extrahepatic organ failure at entry; does not identify exactly the same multiorgan phenotype as EASL-CLIF	Useful where hepatic precipitants predominate and where early liver-failure phenotypes are common
NACSELD	Hospitalized patients with cirrhosis, often in infection-related or general medical admissions	ACLF defined by multiple extrahepatic organ failuresin cirrhosis; simpler binary classification emphasizing bedside identification of high-risk patients	Extrahepatic organ failureand short-term inpatient survival	Primarily in-hospital and 30-day mortality	Simple bedside framework; useful for rapid short-term prognostication; well validated in North American cohorts	Less sensitive than EASL-CLIF; identifies a smaller and often later-stage subgroup; provides less granularity across the ACLF spectrum	Useful for pragmatic short-term risk prediction in hospitalized North American practice, especially infection-related decompensation

**Table 2:** Precipitating events in ACLF: clinical actions for treatment and prevention.

Precipitating Event	Clinical Clues	Immediate Actions (Treatment)	Prevention Strategies
Bacterial infection (SBP, UTI, pneumonia, sepsis)	Fever or hypothermia, encephalopathy, AKI, leukocytosis/leukopenia, ↑CRP/PCT, subtle deterioration	Early broad-spectrum antibiotics, diagnostic paracentesis, cultures (blood/urine/ascites), source control, albumin in SBP, hemodynamic support	SBP prophylaxis (selected patients), early infection screening on admission, antibiotic stewardship, vaccination
Severe alcohol-associated hepatitis	Recent heavy alcohol intake, jaundice, systemic inflammation, renal dysfunction	Exclude infection, consider corticosteroids (selected), nutritional support, early ICU involvement if severe	Alcohol cessation programs, early identification of high-risk drinking, longitudinal addiction care
Gastrointestinal bleeding (variceal/non-variceal)	Hematemesis/melena, anemia, shock, rising urea	Vasoactive drugs (terlipressin), urgent endoscopy, antibiotic prophylaxis, transfusion strategy (restrictive), ICU care if unstable	Non-selective beta-blockers, variceal screening and band ligation, portal pressure control
Drug-induced liver injury/nephrotoxicity	Recent medication change (NSAIDs, antibiotics, contrast), AKI, worsening LFTs	Stop offending drug, supportive care, renal monitoring, avoid nephrotoxins	Medication review, avoid NSAIDs, ACEi, ARB, cautious use of contrast, patient education
Hepatorenal syndrome (often infection-related)	Progressive creatinine rise, low urine sodium, refractory ascites	Albumin + vasoconstrictors (e.g., terlipressin), treat precipitant (often infection), ICU support if needed	Early infection treatment, avoid hypovolemia/nephrotoxins, optimize circulatory status
Ischemia/circulatory failure (shock states)	Hypotension, lactate elevation, AKI, multi-organ dysfunction	Fluid resuscitation (albumin), vasopressors, treat underlying cause (sepsis/bleed), ICU care	Early recognition of sepsis/bleeding, hemodynamic monitoring in high-risk patients
Surgery/invasive procedures	Recent procedure, acute deterioration post-intervention	Early recognition of complications (bleeding, infection), organ support, multidisciplinary care	Careful patient selection, pre-procedure optimization, avoid elective procedures in unstable cirrhosis
Unknown/occult precipitant (~30–40%)	No clear trigger at presentation	Systematic search: repeat cultures, imaging (CT chest/abdomen), medication review, cardiac/pulmonary evaluation	Standardized admission protocols, low threshold for investigation, early reassessment, consider infection as trigger.

**Table 3:** Common severity tools used in decompensated cirrhosis and ACLF.

Tool	Main Variables	Best Use	Main Limitation
MELD/MELD-Na	Bilirubin, INR, creatinine ± sodium	Baseline liver disease severity and allocation systems	Underestimates extrahepatic organ failure and inflammatory state
CLIF-C OF/CLIF-C ACLF	Six organ systems, age, white-cell count	Bedside staging and short-term prognosis in ACLF	More complex; designed for acute decompensation cohorts
NACSELD ACLF score	Number/type of extrahepatic organ failures	Short-term prognostication in hospitalized cirrhosis	Less granular for liver-specific injury and broader disease trajectory

**Table 4:** Key Clinical Conclusions and Practical Implications in ACLF and ESLD.

Domain	Take-Home Message
Natural history	Cirrhosis is dynamic, not unidirectional. Portal hypertension drives decompensation, but etiologic control, prevention of triggers, and functional reserve (including sarcopenia) determine whether patients deteriorate or recompensate.
Precipitating events	ACLF is usually triggered, not spontaneous. Infection, bleeding, alcohol, and drug-induced injury are common drivers—identify and treat precipitants early, as this is often the only reversible component.
Definition and diagnosis	ACLF is defined by organ failure and trajectory, not by bilirubin or MELD alone. Structured assessment (e.g., CLIF-based) and reassessment over 24–72 h are more informative than a single value.
Trajectory over snapshot	Prognosis is dynamic. Failure to improve—or early deterioration—despite initial therapy is more meaningful than admission severity and should accelerate transplant and escalation decisions.
Renal dysfunction (HRS-AKI)	HRS-AKI is frequent, actionable, and time-sensitive. Start protocolized management early (volume, infection control, vasoconstrictors), but interpret response as a bridge—not resolution of ACLF.
Multiorgan support	ICU therapies sustain physiology but rarely reverse ACLF alone. Use them as a bridge to recovery or transplantation—not as open-ended escalation without a defined goal.
Artificial intelligence and prediction	AI and machine-learning models are promising for risk stratification and transplant decision-making, but must complement—not replace—serial clinical assessment and bedside judgment.
Transplantation	Timing matters as much as candidacy. Early referral is essential; even severe ACLF (including grade 3) can have acceptable outcomes if transplanted within the right window.
Systems of care	Outcomes depend on integration. Coordinated pathways and evidence based protocols linking hepatology, ICU, nephrology, infection control, and transplant services outperform isolated decision-making.
Palliative care	Palliative care is not end-of-life care. It should run in parallel with active treatment, addressing symptoms, communication, and decision quality throughout the disease course.
Clinical judgment	The central task is interpretive: identify salvageable biology early, and recognize when further escalation becomes non-beneficial. Both errors—premature nihilism and excessive intervention—carry harm.

## Data Availability

There is no new data collected as this is a narrative review.
